# A Nomogram to Predict Cancer-Specific Survival of Transitional Cell Carcinoma of Ureter After Surgery

**DOI:** 10.3390/medicina61061062

**Published:** 2025-06-09

**Authors:** Der-Shin Ke, Chao-Yu Hsu

**Affiliations:** 1 Department of Neurology, China Medical University Beigang Hospital, Beigang, Yunlin 651012, Taiwan; 2 Department of Medical Education, Ditmanson Medical Foundation, Chia-Yi Christian Hospital, Chia-Yi 600566, Taiwan; 3 Department of Artificial Intelligence and Healthcare Management, Central Taiwan University of Science and Technology, Taichung 406053, Taiwan; 4 Department of General Education, National Chin-Yi University of Technology, Taichung 411030, Taiwan; 5 Department of Senior Citizen Service Management, National Taichung University of Science and Technology, Taichung 404336, Taiwan

**Keywords:** transitional cell carcinoma, ureter, nomogram

## Abstract

*Background and Objectives*: Due to the rare focus on ureteral cancer survival analyses, this study investigates post-surgery cancer-specific survival (CSS) rates, along with prognostic factors affecting these outcomes. It aims to enhance understanding of disease progression and determinants of patient survival and develop a nomogram for reference. *Materials and Methods*: This research undertook a retrospective analysis of ureteral cancer patients who received surgical intervention from 2010 to 2017, utilizing data from the Surveillance, Epidemiology, and End Results database. The primary endpoint was survival, with 1-, 3-, and 5-year CSS rates calculated using the Kaplan–Meier method. Initial univariate Cox proportional hazards analyses identified factors impacting survival, with those yielding a *p*-value under 0.05 progressing to multivariate Cox regression analysis to ascertain significant prognostic indicators. *Results*: The investigation encompassed 2277 patients diagnosed with ureteral cancer. CSS rates at 1, 3, and 5 years post-surgery were observed at 88.2%, 68.1%, and 60.3%, respectively. Multivariate analyses identified age, staging of tumor, node and metastasis, and the application of radiotherapy as significant prognostic indicators for CSS. Based on these factors, a post-surgical nomogram for CSS was developed. *Conclusions*: The survival outcomes for ureteral cancer are not yet satisfactory. Age and stage emerge as pivotal prognostic elements, significantly impacting CSS following surgery. Recognizing these factors is essential for clinicians, as they offer critical insights that inform treatment strategies and patient management.

## 1. Introduction

In an earlier study, Mellemgaard and colleagues [1] observed a significant rise in the incidence of pelvic and ureteral cancers over the period from 1943 to 1988 in Denmark. Specifically, they highlighted that the latter part of this period, between 1983 and 1987, saw an average annual incidence of 55 new cases of ureteral cancer, indicating a notable increase in diagnoses over the examined timeframe. This trend underscores the growing prevalence of this cancer during the mid-20th century. Ramen et al. [2] utilized data from the Surveillance, Epidemiology, and End Results (SEER) database spanning from 1973 to 2005, incorporating a total of 13,800 SEER-registered cases of upper-tract urothelial carcinoma (UTUC). The study observed an upward trend in the overall incidence of UTUC, which escalated from 1.88 to 2.06 cases per 100,000 person-years within the duration of the study. This period also witnessed a corresponding rise in the incidence of ureteral cancer, from 0.69 to 0.91 cases per 100,000 person-years, and a slight reduction in the incidence of renal pelvic cancers, from 1.19 to 1.15 cases per 100,000 person-years. Utilizing the SEER database, two separate studies by Wu et al. [3] and Ma et al. [4] have independently documented a decrease in the incidence rates of UTUC over different periods. Wu et al. observed a marginal but consistent decline in UTUC incidence from 1988 to 2015, with rates slightly decreasing from 1.57 to 1.51 per 100,000 person-years. Complementing this, Ma et al. provided an examination of the period from 2004 to 2019, noting a more pronounced decline in UTUC incidence from 1.46 to 1.27 per 100,000 person-years. Together, these studies underscore a continuing trend of decreasing UTUC incidence, highlighting the value of the SEER database for monitoring cancer epidemiology over extended time frames. In a recent systematic review conducted by Soualhi et al. [5], the incidence of UTUC was reported to exhibit variability across different demographics, including age, sex, race, and other population characteristics. Despite these variations, the authors provided an estimate prevalence for UTUC, which stood at 101.0 per million persons.

In a study conducted by Tan et al. [6] in Taiwan, an examination of patients with ureter urothelial carcinoma revealed a distribution of tumor locations along the ureter. The findings indicated that 54% (38 out of 70) of the tumors were situated in the lower third of the ureter, while 23% (16 out of 70) were located in the upper third, and an equal percentage (23%, 16 out of 70) were found in the middle third. A study conducted by Holmäng et al. [7] in Sweden has demonstrated that the most prevalent site for ureteral tumors is the distal ureter, with findings indicating that 178 out of 331 cases, accounting for 53.8%, were located in this region. Furthermore, the research revealed that proximal ureteral tumors were identified in 71 patients, constituting 21.4% of the cases, whereas tumors situated in the middle segment of the ureter were observed in 56 individuals, representing 16.9% of the total cases examined. These studies contribute to the understanding of tumor distribution in the ureter, highlighting a predominant occurrence in the lower third of the ureter.

Dragicevic et al. [8] conducted a study on pathologically confirmed cases of UTUC observed from 1998 to 2005 in Serbia, focusing on their outcomes over a median follow-up period of 67 months. Despite the relatively small sample size of 114 cases, the study reported a 5-year overall survival (OS) rate of 51.2%. In a research study by Tan et al. [6] from Taiwan, spanning from July 1986 to July 1998, a cohort of 141 patients diagnosed with UTUC was examined. This cohort comprised 71 individuals with renal pelvic and 70 with ureteral urothelial carcinoma. The investigation aimed to analyze the outcomes of these patients, culminating in an overall 5-year survival rate of 48%. In a recent study by Gao et al. [9], the survival outcomes of 401 Chinese patients with UTUC were assessed based on risk categories defined by the number of independent risk factors present. The study delineated three risk groups: low-risk (0–1 factors), medium-risk (2 factors), and high-risk (3–5 factors). It was found that in the high-risk group, OS rates at 1, 3, and 5 years were 74.1%, 51.3%, and 42.3%, respectively, while cancer-specific survival (CSS) rates for the same intervals were 76.1%, 54.7%, and 46.4%, respectively. These findings contribute to the body of knowledge on the prognosis of UTUC, delineating survival outcomes in the population over a specified period.

Several studies have proposed nomograms to predict outcomes in patients with UTUC. For instance, Wu et al. [10] and Luo et al. [11] developed nomograms to predict extra-urothelial recurrence following radical nephroureterectomy. Tian et al. [12] established a model to estimate OS, while Jeldres et al. [13], utilizing data from the SEER database, constructed a nomogram to predict CSS. More recently, Yates et al. [14] also developed a nomogram for CSS prediction. Notably, the latter study enrolled only 667 patients, including 397 in the development cohort and 270 in the validation cohort. Given the relative paucity of survival analyses specifically focused on ureteral cancer, the present study aims to investigate CSS following surgical treatment. Furthermore, we seek to identify prognostic factors associated with CSS to improve the understanding of disease progression and survival outcomes. Based on these findings, a clinically applicable nomogram will be developed as a prognostic tool for reference in patient management.

## 2. Materials and Methods

### 2.1. Data Source

This study conducted a retrospective analysis of patients diagnosed with ureteral cancer who underwent surgical treatment between 2010 and 2017. The data were extracted from the SEER database, utilizing version 8.4.3. This database is recognized for its extensive collection of cancer incidence and survival data from population-based cancer registries of the U.S. population, making it a valuable resource for epidemiological and health outcomes research. This study received ethical approval from the Ethics Committee of “Ditmanson Medical Foundation Chia-Yi Christian Hospital” (IRB2024027). Owing to the nature of the patient data employed, which was derived from the SEER and is anonymized, the requirement for written informed consent from study participants was exempted.

### 2.2. Study Population

The inclusion criteria were specifically designed to target patients with ureteral cancer who had undergone surgery, as surgical reports provide the most detailed and definitive pathological information. Only those cases with complete pathological reports were considered, ensuring the accuracy and reliability of the cancer staging and grading information. Several exclusion criteria were applied to refine the study population and enhance the validity of the findings. These criteria included patients with bilateral tumors, unclear histology, follow-up less than 1 month, unknown grade, and indeterminate Tumor (T), Node (N), Metastasis (M) staging, which precluded precise stage determination. Additionally, cases with ambiguous survival status were excluded to ensure the integrity of survival analyses.

### 2.3. Study Variables

The study collected a comprehensive set of variables to investigate the demographic, pathological, and treatment-related factors influencing ureteral cancer outcomes. Demographic Information: gender and race (categorized as white and others), along with age at diagnosis, stratified into 4 groups (<65, 65–74, 75–84, ≥85). Pathological characteristics: histology (specifically, transitional cell carcinoma (TCC) and papillary TCC), grade (well differentiated, moderately differentiated, poorly differentiated, and undifferentiated), and the 7th American Joint Committee on Cancer (AJCC) stage based on TNM classification. Treatment modalities: consideration of radiotherapy and chemotherapy as part of the treatment regimen.

### 2.4. Statistical Analysis

Survival outcomes were the primary focus of this study. The Kaplan–Meier method was employed to calculate the 1-, 3-, and 5-year CSS. CSS was defined as the interval from the date of diagnosis to death attributed to ureteral cancer, based on SEER’s variables “survival months” and “SEER cause-specific death classification”. This method facilitated the evaluation of survival probabilities over time, providing a clear picture of long-term outcomes. The survival curves generated allowed for the assessment of the impact of various demographic, pathological, and treatment variables on survival rates. To identify factors significantly associated with survival outcomes, a univariate Cox proportional hazards model was initially used. Variables with a *p*-value less than 0.05 in the univariate analysis were considered statistically significant and were subsequently included in a multivariate Cox regression analysis. This approach enabled the identification of independent prognostic factors, offering insights into the variables that significantly affect CSS in patients with ureteral cancer. Subsequently, we divided the patients into two groups, with a ratio of 7:3, a training cohort and a validation cohort. The prognostic factors derived from the Cox regression were then analyzed. If the concordance index (C-index) exceeded 0.7, indicating suitability for model development, a nomogram was constructed. To validate the reliability of the nomogram, the model’s predictive performance was assessed using operating characteristic curve (ROC), calibration, and decision curve analysis (DCA). The statistical analyses were performed using the R software version 4.3.2. A *p*-value of less than 0.05 was considered indicative of statistical significance throughout the study.

## 3. Results

**Figure 1:**
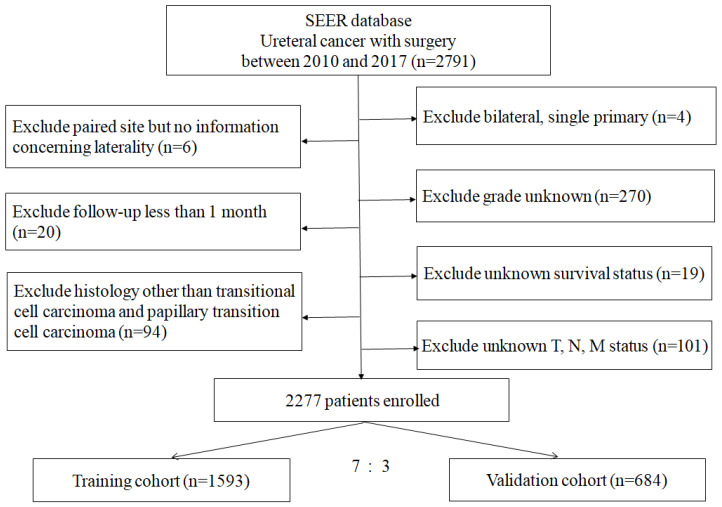
Flowchart of the patients’ selection.

The study included a total of 2277 patients, with 1138 having tumors on the right side and 1139 on the left side. The median duration of follow-up for these patients was 49.0 months, with a range extending from 1 to 143 months. The selection process of patients is detailed in Figure 1, presenting a structured flowchart. Table 1 presents the baseline characteristics of patients in the total, training, and validation cohorts. The cohorts consist of 2277 patients, with 63.8% male and 36.2% female. The racial composition is predominantly White (87.2%). Age distribution reveals that 20.3% are under 65, 34.0% are between 65 and 74, 33.7% are between 75 and 84, and 12.0% are 85 or older. Histologically, patients are almost evenly split between TCC (49.9%) and papillary TCC (50.1%). Grade distribution shows a higher prevalence of undifferentiated tumors (62.3%), followed by poorly (21.3%), moderately (12.2%), and well-differentiated (4.3%) tumors. The stage at diagnosis is primarily Stage I (33.0%) and Stage III (27.8%). Tumor characteristics indicate a higher frequency of T1 (33.9%) and T3 (34.8%) tumors. Node involvement is mostly absent (89.9%), and metastasis is rare (3.5%). Treatment modalities indicate that the majority did not receive radiotherapy (93.9%) or chemotherapy (75.9%). The *p*-values across variables suggest no significant differences between the training and validation cohorts, affirming the comparability of these groups for subsequent analyses. We also observed that lymphadenectomy was performed in 811 patients, with 347 patients undergoing removal of one to three lymph nodes and 464 patients having four or more lymph nodes removed. Statistical analysis revealed no significant difference between patients who underwent lymphadenectomy and those who did not (*p* = 0.49). Among patients with Stage II or higher, the lymphadenectomy group still did not exhibit a significant difference in CSS at 1, 3, and 5 years compared to the non-lymphadenectomy group (85.5%, 57.4%, and 51.5% vs. 83.6%, 58.9%, and 48.5%, *p* = 0.33, respectively).

The CSS at 1, 3, and 5 years was 88.2%, 68.1%, and 60.3%, respectively. Table 2 presents the results of univariate and multivariate analyses of CSS in the training cohort. In terms of sex, females showed a hazard ratio (HR) of 1.15 (*p* = 0.079) in univariate analysis, indicating a non-significant trend towards higher risk compared to males. Race did not significantly affect survival (HR = 1.06, *p* = 0.645). Age was a significant factor, with increased risk observed in older age groups: HR 1.38 (*p* = 0.009) for ages 65–74, HR 1.79 (*p* < 0.001) for ages 75–84, and HR 2.51 (*p* < 0.001) for ages 85 and older in univariate analysis. These findings were consistent in multivariate analysis. Histology indicated that papillary TCC had a significantly lower risk in univariate analysis (HR = 0.53, *p* < 0.001), although this was not significant in multivariate analysis. Higher tumor grade was associated with increased risk, but only poorly differentiated tumors showed a significant increase in univariate analysis (HR = 2.76, *p* < 0.001). Stage and tumor characteristics were strong predictors of survival. Advanced stages (III and IV) and higher T stages (T3 and T4) were associated with significantly increased risk. Node involvement and metastasis were also significant predictors, with higher HRs for N2–3 and M1 categories. Radiotherapy and chemotherapy were significant in univariate analysis, but only radiotherapy remained significant in multivariate analysis (HR = 1.66, *p* < 0.001).

**Figure 2:**
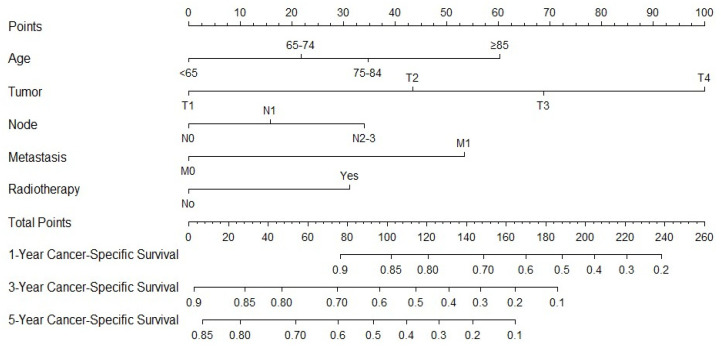
Nomogram to predict 1-, 3-, and 5-year cancer-specific survival rates in patients with transitional cell carcinoma and papillary transitional cell carcinoma after surgery.

**Figure 3:**
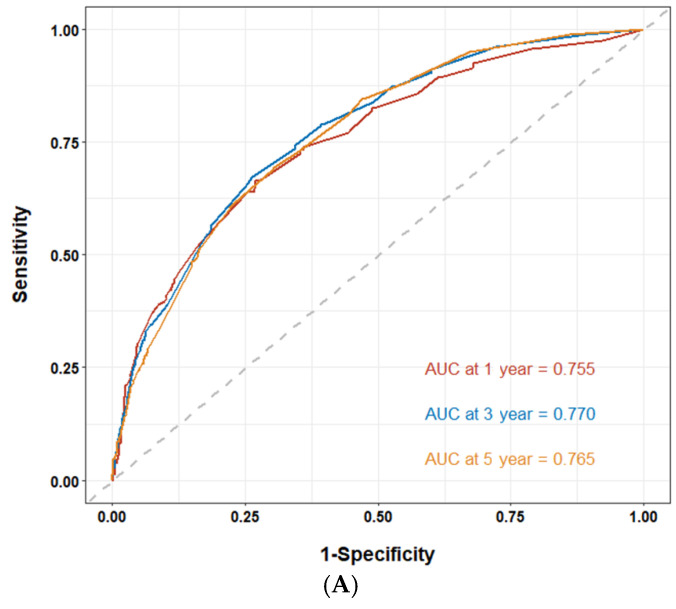

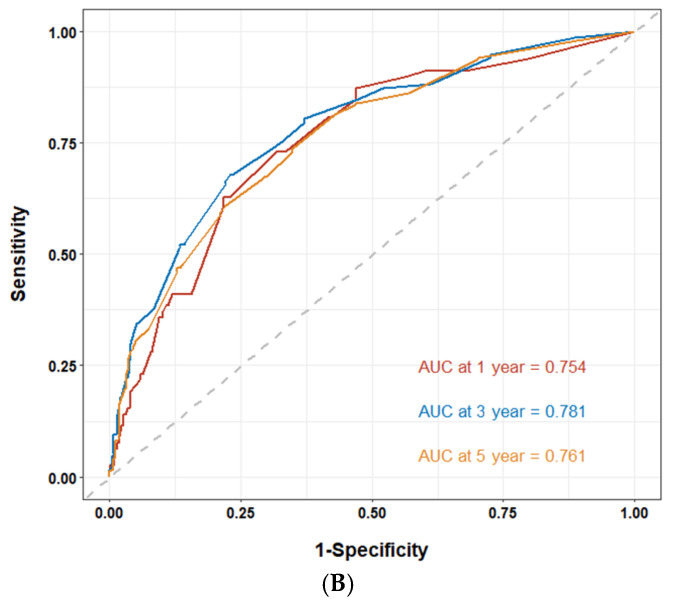
Analysis of the receiver operating characteristic (ROC) curve for the nomogram in predicting prognosis in the training and validation cohorts. The area under the ROC curve (AUC) for 1-, 3-, and 5-year cancer-specific survival in the training (n = 1593) (**A**) and validation cohorts (n = 684) (**B**).

**Figure 4:**
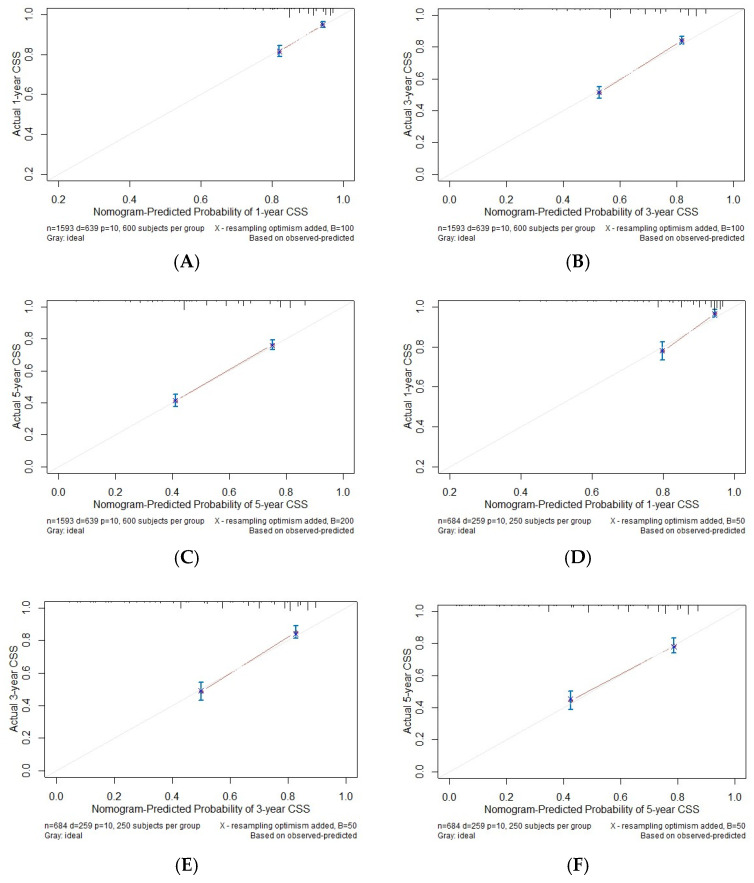
The calibration plots for predicting cancer-specific survival (CSS) in the training cohort at 1 (**A**), 3 (**B**), and 5 years (**C**) and in the validation cohort at 1 (**D**), 3 (**E**), and 5 years (**F**). Actual CSS is plotted on the y-axis; nomogram-predicted probability of CSS is plotted on the x-axis.

**Figure 5:**
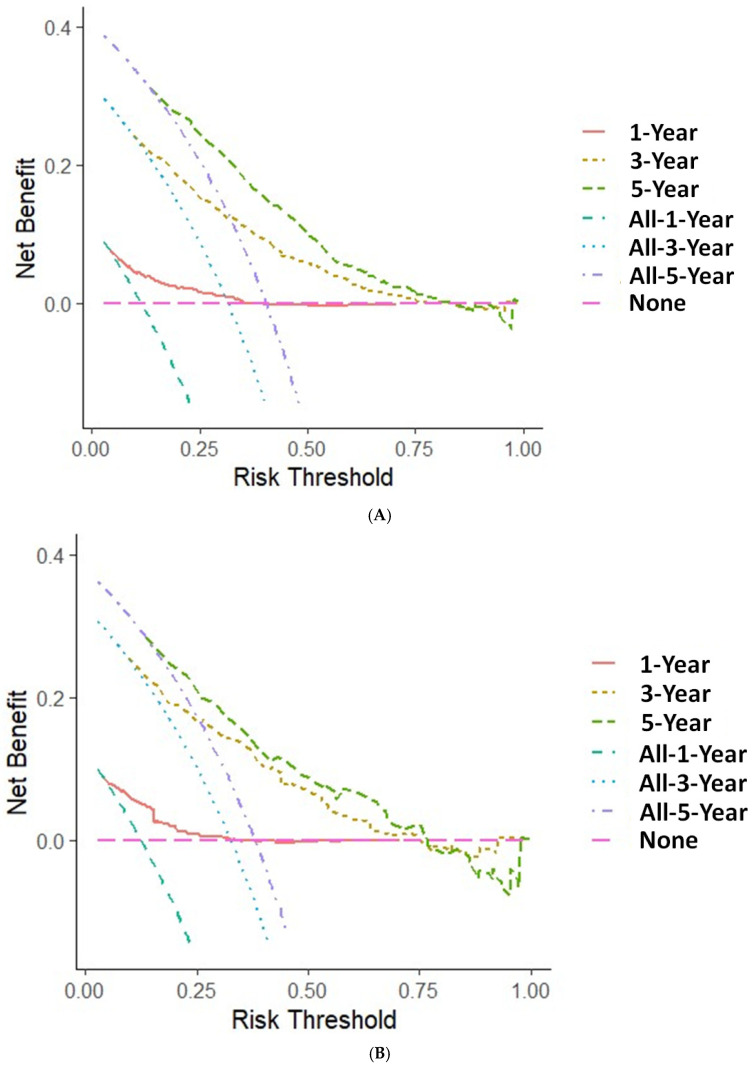
Decision curve analysis of the nomogram for predicting 1-, 3- and 5-year cancer-specific survival in the training (**A**) and validation cohorts (**B**). The x-axis is the threshold probability, and the y-axis is the net benefit.

Since the staging is derived from T, N, and M classifications, we employed these parameters in our multivariate analysis. Our findings indicate that age, T, N, M, and radiotherapy are significant factors influencing CSS. The c-index calculated from these factors is 0.72, demonstrating the suitability of the established model, which is illustrated in Figure 2. Using ROC analysis to validate the model, we observed that the 1-, 3-, and 5-year area under the ROC curve (AUC) in the training cohort is 0.755, 0.770, and 0.765, respectively. In the validation cohort, the 1-, 3-, and 5-year AUCs are 0.754, 0.781, and 0.761, respectively (Figure 3). Figure 4 presents the 1-, 3-, and 5-year calibration for both the training and validation cohorts. Figure 5 displays the DCA, providing evidence of the reliability of the predictive model.

## 4. Discussion

In the conducted study, the observed CSS of ureteral cancer at 1, 3, and 5 years was 88.2%, 68.1%, and 60.3%, respectively. The investigation further identified age, T, N, M and the application of radiotherapy as significant prognostic factors influencing CSS.

Multiple investigators have reported outcomes following ureteral cancer treatment. Lehmann et al. [15] examined 145 consecutive patients treated with partial ureterectomy or nephroureterectomy and demonstrated that 5-year disease-specific survival varied substantially by pathological stage, ranging from 96.1% for pTa tumors to 28.6% for pT4 lesions. Xia et al. [16] utilized the SEER database to evaluate survival outcomes following segmental ureterectomy combined with chemotherapy in patients with high-grade non-metastatic ureteral cancer. Among 1757 patients, the reported 1-, 3- and 5-year OS rates were 82.8%, 55.6%, and 42.8%, respectively. However, as this study focused on OS rather than CSS, direct comparison with our findings is limited. Subsequently, Ding et al. [17] analyzed SEER data from 1910 patients diagnosed with primary TCC of the ureter between 2004 and 2013. They reported 5-year OS and CSS rates of 41.8% and 54.3%, respectively. The 5-year CSS rate was marginally lower than that observed in the present study (60.3%); this discrepancy may be attributable to differences in median follow-up duration (49.0 months in the current study versus 78 months in their analysis).

A study conducted by Ferro et al. [18], with a cohort of 776 patients with clinically non-metastatic UTUC, who underwent radical nephroureterectomy across 21 academic hospital centers between 2005 and 2021, was analyzed. The findings revealed that CSS was statistically significantly correlated with age, identifying it as an independent prognostic factor (*p* = 0.0001). In a systematic review and meta-analysis, the threshold for advanced age was established at 70 years. Consistent with this determination, Ye et al. [19] reported findings that corroborate the association between advanced age and decreased CSS and OS. Specifically, advanced age was linked with an inferior OS, as evidenced by a pooled HR of 1.55, and a diminished CSS, with a pooled HR of 1.37. Our findings also indicate that age constitutes a significant prognostic factor for CSS. An increase in age is associated with a heightened risk, underscoring the pivotal role of age in predicting survival outcomes subsequent to surgical intervention for ureteral cancer.

Claps et al. [20] analyzed 1082 patients who underwent radical cystectomy for cT1-4aN0M0 urothelial bladder cancer, finding that 72.5% had pure urothelial carcinoma, while 27.5% exhibited variant histologies. Martini et al. [21] reported that variant histology bladder cancer is associated with a higher risk of recurrence compared to pure urothelial carcinoma, with patients exhibiting variant histology having a significantly lower recurrence-free survival rate (30% vs. 51% at 10 years, *p* < 0.001) and a shorter median time to recurrence (88 vs 123 months, *p* < 0.01). In the present study, we focused exclusively on TCC of the ureter, without comparing other histological types. We reported CSS for ureteral TCC and developed a nomogram for reference.

The study by Brown et al. [22] suggested that the histologic grade from diagnostic biopsies for UTUC could predict the disease’s pathologic stage. In our study with a large sample size, we found that Grade III and IV histologic diagnoses are significantly correlated with CSS in univariate Cox analysis. However, this phenomenon was not observed in the multivariate analysis, likely due to the high proportion of undifferentiated cases (62.3%). Kim et al. [23] identified lymphovascular invasion and pathological T stage as important predictors of CSS in patients with localized UTUC. Specifically, those with pathologic T3 and positive lymphovascular invasion had significantly poorer disease-specific survival compared to patients with lower risk. Tai et al. [24] explored the impact of tumor location on oncologic outcomes in patients with UTUC, finding no difference in outcomes between pathological T3 and T2 of renal pelvic carcinoma. However, pathological T3 ureteral cancer was associated with significantly shorter CSS and increased risks of cancer-specific death and overall mortality compared to pathological T2 diseases. The authors recommended close follow-up and consideration of adjuvant chemotherapy for patients with pathological T3 ureteral cancer. In our study, T, N, M, and stage were associated with CSS in univariate Cox analysis. Given that the AJCC stage encompasses T, N, and M, we employed these variables in the multivariate Cox analysis. The analysis revealed that T, N, and M are significant predictors of CSS.

Dominguez-Escrig et al. [25] conducted a systematic review of the literature to evaluate the impact of lymphadenectomy in patients with UTUC. Their findings indicated that lymphadenectomy improves CSS in patients with high-stage (≥pT2) and reduces the risk of local recurrence. While lymphadenectomy enhances survival in patients with high-stage renal pelvic tumors, its impact on ureteral tumors remains uncertain. Our results did not demonstrate a significant benefit of lymphadenectomy for ureteral TCC. Although patients with Stage II or higher who underwent lymphadenectomy showed a marginally better 5-year CSS compared to those who did not (51.5% vs. 48.5%), the difference was not statistically significant. However, we cannot draw definitive conclusions from these results due to the selection bias inherent in our study, as we only enrolled patients who underwent surgery, which implies a well-selected group. Consequently, only 10% of these patients had positive lymph nodes. According to the report by Pandolfo et al. [26], current clinical guidelines concur in recommending radical nephroureterectomy with bladder cuff excision and lymphadenectomy as the standard therapeutic approach for the management of high-risk non-metastatic UTUC. To better assess the true prognostic and therapeutic value of lymphadenectomy, future studies should consider prospective designs, including randomized controlled trials where feasible. Additionally, large-scale observational studies utilizing methods such as propensity score matching or inverse probability of treatment weighting could help mitigate confounding and provide more robust estimates of treatment effect in real-world populations.

In a meta-analysis by Zalay et al. [27] involving 20 studies, it was determined that adjuvant radiotherapy following nephroureterectomy for patients with locally advanced UTUC significantly reduces the risk of locoregional recurrence. However, it was noted that this intervention does not enhance the OS. Correspondingly, research conducted by Chen et al. [28] corroborated these findings, highlighting no substantial difference in OS between patients with UTUC who received radiotherapy and those who did not. Yet, for individuals with T3 or T4 staging of the disease, radiotherapy was observed to potentially improve OS, indicating a stage-specific benefit of this treatment modality. Additionally, Li et al. [29] presented evidence suggesting that while radiotherapy does not increase the CSS across all patients with UTUC, it does significantly benefit those with high-risk factors such as multifocality, pathological T3 or T4 staging, Grade III tumors, and positive lymph node status. Our findings reinforce the prognostic significance of radiotherapy in CSS among patients with ureteral cancer. While radiotherapy may offer therapeutic benefits, its associated risks warrant careful consideration. Acute and chronic adverse effects—such as radiation-induced fibrosis, tissue damage, and injury to adjacent organs—can compromise quality of life and long-term outcomes. Moreover, the observed association between radiotherapy and poorer prognosis may, in part, reflect confounding by indication. Patients undergoing radiotherapy often present with more advanced disease, incomplete resection, or other unfavorable clinical features. These underlying conditions may account for worse survival outcomes, rather than radiotherapy itself. This potential selection bias must be acknowledged in retrospective studies. Further prospective research is needed to clarify the true impact of radiotherapy in advanced ureteral cancer and to inform evidence-based treatment strategies.

The SEER database of the United States provides invaluable information for cancer research, offering extensive data on cancer incidence, survival, and prevalence. However, it is not without its limitations, particularly in the context of ureteral cancer research. First, the SEER database lacks detailed information regarding the duration of the preoperative waiting period, the specific type of surgery performed, bladder cuff excision, and the presence of bladder tumors. These factors may significantly impact postoperative outcomes. Veccia et al. [30] conducted a study examining the outcomes of robotic radical nephroureterectomy versus laparoscopic radical nephroureterectomy. Their findings indicated that the overall complication rate was higher for laparoscopic radical nephroureterectomy, whereas robotic radical nephroureterectomy was associated with a reduced length of hospital stay. They concluded that the adoption of robotic surgery might enhance the likelihood of achieving a “tetrafecta” outcome. However, it is noteworthy that the specific surgical types were not recorded in the SEER database. Second, significant limitations include the database’s lack of specificity regarding the surgical margin and lymph node density. These omissions hinder the ability to accurately assess the severity and prognosis of ureteral cancer, which is essential for tailoring treatment strategies and predicting patient outcomes. However, in constructing the nomogram, we utilized the T, N, and M classifications instead of the overall stage to provide a more detailed analysis, aiming to minimize bias. Third, the SEER database lacks granular details regarding treatment modalities, including timing (e.g., adjuvant vs. neoadjuvant), dosage, specific regimens, and therapeutic intent. Although it captures whether patients received radiotherapy or chemotherapy, the absence of more comprehensive treatment data precludes a nuanced analysis of how different protocols may influence survival outcomes. This inherent limitation, common to SEER-based studies, may affect the interpretability of treatment-related prognostic associations. Nevertheless, the inclusion of these variables still provides meaningful, population-level insights into the relationship between treatment exposure and CSS. Fourth, the SEER database does not include important clinical variables such as comorbidities, performance status, or disease recurrence. The absence of these factors introduces the potential for selection bias and limits our ability to control for key prognostic determinants. To mitigate this limitation, our analysis focused on CSS rather than OS, as CSS is less likely to be confounded by non-cancer-related mortality in the absence of comorbidity data. Nonetheless, we acknowledge that the lack of data on functional status and recurrence remains a significant constraint in fully assessing patient prognosis. Fifth, the SEER database does not capture lifestyle-related variables, such as smoking status, which may significantly influence patient prognosis. Previous studies, including Kumar et al. [31], have reported a strong association between tobacco use and the tumorigenesis of UTUC. Smoking has also been correlated with poorer survival outcomes, and elevated risk of recurrence. Additionally, although the nomogram demonstrated acceptable discriminative performance with AUC values ranging from 0.75 to 0.78, the calibration plots revealed slight deviations at higher predicted probability levels. This discrepancy may be attributed to potential overfitting of the model or the limited number of high-risk patients within this range, which could reduce the accuracy of predictions in those subgroups. Furthermore, as this study was based on retrospective data from a population-based registry, prospective external validation is warranted to confirm the model’s generalizability and robustness in other clinical settings. Even with its limitations, SEER’s extensive data is a valuable resource for evidence-based medicine, offering significant insights that can guide clinical decision-making and contribute to the improvement of ureteral cancer care.

## 5. Conclusions

The survival rate for ureteral cancer remains suboptimal. Age and stage are critical prognostic factors that must be particularly considered post-surgery, as they influence CSS. These prognostic factors and post-surgical nomogram serve as valuable references for clinicians when treating patients.

## Figures and Tables

**Table 1:** Characteristics of the total, training, and validation cohorts.

Variables		Total	Training Cohort	Validation Cohort	*p*-Value
Sex	Male	1452 (63.8%)	1019 (64.0%)	433 (63.3%)	0.7628
	Female	825 (36.2%)	574 (36.0%)	251 (36.7%)	
Race	White	1986 (87.2%)	1390 (87.3%)	596 (87.1%)	0.9362
	Others	291 (12.8%)	203 (12.7%)	88 (12.9%)	
Age	<65	462 (20.3%)	316 (19.8%)	146 (21.3%)	0.7334
	65–74	774 (34.0%)	544 (34.1%)	230 (33.6%)	
	75–84	767 (33.7%)	535 (33.6%)	232 (33.9%)	
	≥85	274 (12.0%)	198 (12.4%)	76 (11.1%)	
Histology	Transitional cell carcinoma	1137 (49.9%)	810 (50.8%)	327 (47.8%)	0.1834
	Papillary transitional cell carcinoma	1140 (50.1%)	783 (49.2%)	357 (52.2%)	
Grade	Well	97 (4.3%)	71 (4.5%)	26 (3.8%)	0.0542
	Moderately	277 (12.2%)	196 (12.3%)	81 (11.8%)	
	Poorly	485 (21.3%)	361 (22.7%)	124 (18.1%)	
	Undifferentiated	1418 (62.3%)	965 (60.6%)	453 (66.2%)	
Stage	I	751 (33.0%)	529 (33.2%)	222 (32.5%)	0.5890
	II	587 (25.8%)	408 (25.6%)	179 (26.2%)	
	III	633 (27.8%)	451 (28.3%)	182 (26.6%)	
	IV	306 (13.4%)	205 (12.9%)	101 (14.8%)	
Tumor	T1	772 (33.9%)	542 (34.0%)	230 (33.6%)	0.3736
	T2	633 (27.8%)	430 (27.0%)	203 (29.7%)	
	T3	793 (34.8%)	569 (35.7%)	224 (32.7%)	
	T4	79 (3.5%)	52 (3.3%)	27 (3.9%)	
Node	N0	2048 (89.9%)	1437 (90.2%)	611 (89.3%)	0.2108
	N1	117 (5.1%)	74 (4.6%)	43 (6.3%)	
	N2–3 (only 5 patients with N3)	112 (4.9%)	82 (5.1%)	30 (4.4%)	
Metastasis	M0	2198 (96.5%)	1539 (96.6%)	659 (96.3%)	0.7513
	M1	79 (3.5%)	54 (3.4%)	25 (3.7%)	
Radiotherapy	No	2137 (93.9%)	1493 (93.7%)	644 (94.2%)	0.6957
	Yes	140 (6.1%)	100 (6.3%)	40 (5.8%)	
Chemotherapy	No	1729 (75.9%)	1208 (75.8%)	521 (76.2%)	0.8628
	Yes	548 (24.1%)	385 (24.2%)	163 (23.8%)	

**Table 2:** Univariate and multivariate analysis of cancer-specific survival in the training cohort.

Variables		Number (%)	HR (Univariable)	HR (Multivariable)
Sex	Male	1019 (64.0%)		
	Female	574 (36.0%)	1.15 (0.98–1.35, *p* = 0.079)	
Race	White	1390 (87.3%)		
	Others	203 (12.7%)	1.06 (0.84–1.33, *p* = 0.645)	
Age	<65	316 (19.8%)		
	65–74	544 (34.1%)	1.38 (1.08–1.75, *p* = 0.009)	1.42 (1.12–1.81, *p* = 0.004)
	75–84	535 (33.6%)	1.79 (1.42–2.27, *p* < 0.001)	1.76 (1.38–2.24, *p* < 0.001)
	≥85	198 (12.4%)	2.51 (1.90–3.32, *p* < 0.001)	2.66 (1.99–3.56, *p* < 0.001)
Histology	Transitional cell carcinoma	810 (50.8%)		
	Papillary transitional cell carcinoma	783 (49.2%)	0.53 (0.45–0.62, *p* < 0.001)	0.85 (0.71–1.01, *p* = 0.057)
Grade	Well	71 (4.5%)		
	Moderately	196 (12.3%)	0.88 (0.49–1.58, *p* = 0.669)	0.78 (0.43–1.41, *p* = 0.414)
	Poorly	361 (22.7%)	2.76 (1.66–4.62, *p* < 0.001)	1.44 (0.85–2.45, *p* = 0.171)
	Undifferentiated	965 (60.6%)	2.62 (1.59–4.31, *p* < 0.001)	1.44 (0.86–2.40, *p* = 0.167)
Stage	I	529 (33.2%)		
	II	408 (25.6%)	2.08 (1.63–2.65, *p* < 0.001)	
	III	451 (28.3%)	3.54 (2.83–4.44, *p* < 0.001)	
	IV	205 (12.9%)	5.97 (4.65–7.68, *p* < 0.001)	
Tumor	T1	542 (34.0%)		
	T2	430 (27.0%)	2.16 (1.71–2.73, *p* < 0.001)	1.81 (1.42–2.31, *p* < 0.001)
	T3	569 (35.7%)	3.75 (3.03–4.64, *p* < 0.001)	2.65 (2.09–3.37, *p* < 0.001)
	T4	52 (3.3%)	7.98 (5.50–11.56, *p* < 0.001)	4.53 (3.04–6.76, *p* < 0.001)
Node	N0	1437 (90.2%)		
	N1	74 (4.6%)	2.25 (1.66–3.06, *p* < 0.001)	1.24 (0.89–1.72, *p* = 0.203)
	N2-3	82 (5.1%)	2.96 (2.25–3.89, *p* < 0.001)	1.71 (1.26–2.31, *p* = 0.001)
Metastasis	M0	1539 (96.6%)		
	M1	54 (3.4%)	5.05 (3.72–6.86, *p* < 0.001)	2.48 (1.76–3.49, *p* < 0.001)
Radiotherapy	No	1493 (93.7%)		
	Yes	100 (6.3%)	2.39 (1.87–3.06, *p* < 0.001)	1.66 (1.28–2.16, *p* < 0.001)
Chemotherapy	No	1208 (75.8%)		
	Yes	385 (24.2%)	1.49 (1.26–1.76, *p* < 0.001)	0.95 (0.78–1.15, *p* = 0.579)

## Data Availability

The data utilized and analyzed in this study can be accessed through an open database, namely the Surveillance, Epidemiology, and End Results program, available at https://seer.cancer.gov/ (accessed on 15 July 2024).

## List of Abbreviations

AJCC, American Joint Committee on Cancer; AUC, area under the ROC curve; C-index, concordance index; CSS, cancer-specific survival; DCA, decision curve analysis; HR, hazard ratio; M, metastasis; N, node; OS, overall survival; ROC, operating characteristic curve; SEER, Surveillance, Epidemiology, and End Results; T, tumor; TCC, transitional cell carcinoma; UTUC, upper-tract urothelial carcinoma.
